# The Effect of Tryptophan-to-Tyrosine Mutation at Position 61 of the Nonstructural Protein of Severe Fever with Thrombocytopenia Syndrome Virus on Viral Replication through Autophagosome Modulation

**DOI:** 10.3390/ijms25126394

**Published:** 2024-06-10

**Authors:** Ji-Young Park, Amal Senevirathne, Khristine Kaith S. Lloren, John Hwa Lee

**Affiliations:** College of Veterinary Medicine, Jeonbuk National University, Iksan 54596, Republic of Korea

**Keywords:** SFTSV, NSs, NP, replication, autophagosome

## Abstract

In our prior investigations, we elucidated the role of the tryptophan-to-tyrosine substitution at the 61st position in the nonstructural protein NSsW61Y in diminishing the interaction between nonstructural proteins (NSs) and nucleoprotein (NP), impeding viral replication. In this study, we focused on the involvement of NSs in replication via the modulation of autophagosomes. Initially, we examined the impact of NP expression levels, a marker for replication, upon the infection of HeLa cells with severe fever thrombocytopenia syndrome virus (SFTSV), with or without the inhibition of NP binding. Western blot analysis revealed a reduction in NP levels in NSsW61Y-expressing conditions. Furthermore, the expression levels of the canonical autophagosome markers p62 and LC3 decreased in HeLa cells expressing NSsW61Y, revealing the involvement of individual viral proteins on autophagy. Subsequent experiments confirmed that NSsW61Y perturbs autophagy flux, as evidenced by reduced levels of LC3B and p62 upon treatment with chloroquine, an inhibitor of autophagosome–lysosome fusion. LysoTracker staining demonstrated a decrease in lysosomes in cells expressing the NS mutant compared to those expressing wild-type NS. We further explored the mTOR-associated regulatory pathway, a key regulator affected by NS mutant expression. The observed inhibition of replication could be linked to conformational changes in the NSs, impairing their binding to NP and altering mTOR regulation, a crucial upstream signaling component in autophagy. These findings illuminate the intricate interplay between NSsW61Y and the suppression of host autophagy machinery, which is crucial for the generation of autophagosomes to facilitate viral replication.

## 1. Introduction

Severe fever with thrombocytopenia syndrome (SFTS) is caused by the SFTS virus (SFTSV), which is a member of the genus *Bandavirus* and the family *Phenuiviridae*. Patient zero with an SFTSV infection was found in 2009 in China, followed by rapid dispersal of SFTSV in other countries, including Korea, Japan, Vietnam, Myanmar, and Thailand, between 2010 and 2020 [1,2,3]. A virus species with similarity to SFTSV was identified in the United States in 2012 and named the Heartland virus (HRTV) [4]. The emergence of the virus in different parts of the world within a short period has drawn the attention of healthcare professionals and researchers worldwide, and the viral agent has been recognized as a high-priority research candidate. The disease is characterized by thrombocytopenia, leukocytopenia, high fever, and gastrointestinal discomfort [5]. Currently, SFTSV is reported to have 5–30% mortality in East Asian countries [6]. Tick species such as *Haemaphysalis longicornis* are recognized as competent vectors for viral transmission, while occasional incidents of patient-to-patient transmission also have been reported [7]. Evidence suggests that transmission via blood, body fluids, and aerosols could cause viral spread from patient to patient [8]. Despite the severity of SFTS, there are currently no specific antiviral medications or vaccines available for its prevention or treatment. Hence, control of initial infection is of primary importance. Furthermore, our understanding of the pathogenesis of SFTSV infection and the host immune responses to the virus remains incomplete. Clinically, one notable characteristic of SFTSV infection is the suppression of interferon (IFN) production in affected individuals, indicating the effective evasion of the innate immune response by the virus [9]. This immune evasion likely contributes to the pathogenesis of SFTS by enabling viral replication and systemic dissemination. Hence, for the development of therapeutic approaches, it is essential to understand the precise mechanisms underlying the suppression of IFN production and the modulation of host immune responses.

The genome of SFTSV consists of three negative-stranded RNA segments, namely large (L), medium (M), and small (S). Among these segments, the S segment encodes two essential viral proteins, nucleoprotein (NP) and nonstructural proteins (NSs) [10]. NP plays a crucial role in viral replication by encapsulating the viral genome into ribonucleoprotein complexes (RNPs) and modulating host cellular processes, including autophagy [10]. In contrast, NSs act as a putative virulence factor that promotes viral replication and dissemination by forming viroplasm-like structures and inhibiting host immune responses. Autophagy, a highly conserved cellular process, plays a critical role in maintaining cellular homeostasis and regulating immune responses, including antiviral defense mechanisms [11]. During viral infections, autophagy can have both pro- and antiviral effects, depending on the context and the specific virus involved. In SFTSV infection, deregulation of autophagy has been implicated in the viral pathogenesis, with increased autophagy activity correlating with enhanced viral replication and dissemination [12]. Several viruses, including hepatitis C virus (HCV), Epstein–Barr virus (EBV), dengue virus, and poliovirus, as well as SFTSV, have been shown to manipulate cellular autophagy pathways to promote viral replication and survival [13,14,15]. By modulating autophagy, viruses can subvert host defense mechanisms and create a favorable environment for their replication and dissemination. However, the specific interactions between cellular autophagy pathways and SFTSV proteins remain poorly characterized.

In this study, we investigate the impact of NP and NSs, the major proteins involved in SFTSV virulence, on autophagosome formation. By elucidating the mechanisms underlying the modulation of autophagy by SFTSV proteins, we seek to gain insights into the pathogenesis of SFTSV infection and identify potential targets for therapeutic intervention. Understanding the interplay between SFTSV and the host autophagy machinery may pave the way for the development of new antiviral strategies to combat SFTS and other emerging infectious diseases caused by related viruses.

## 2. Results

### 2.1. NS Expression Induces the Formation of Autophagosomes and Increases Replication

**Figure 1:**
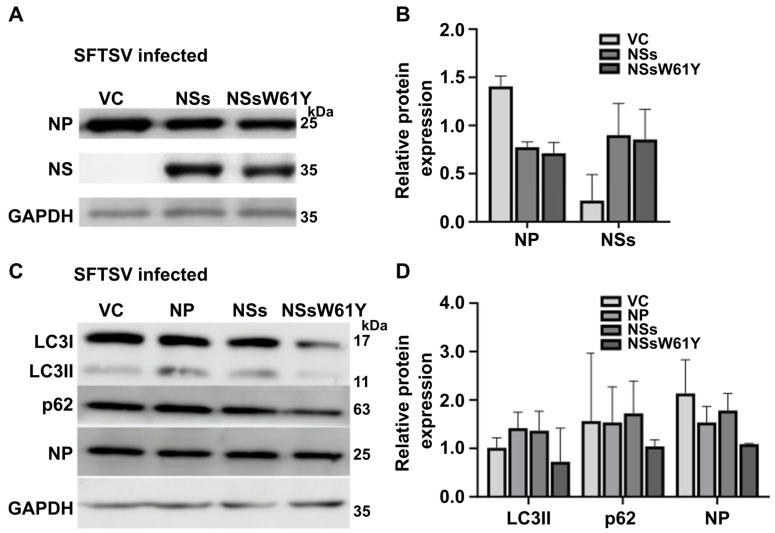
NSsW61Y regulation of viral replication and autophagosome formation. (**A**,**B**) HeLa cells were transfected with plasmids encoding NSs and NSsW61Y for 24 h, followed by infection with SFTSV at an MOI of 0.5 for 24 h. Cell lysates were collected using RIPA lysis buffer and analyzed by Western blot using the indicated antibodies. Quantitative results of expression of NP and NSs were normalized by GAPDH using Image J software. (**C**,**D**) NSsW61Y-transfected cells exhibited downregulation of LC3 and p62 levels, as well as NP expression levels. Quantitative results of expression level of LC3II, P62, and NSs were normalized by GAPDH using Image J software. Data from independent experiments are presented as the mean intensity of the protein band ±SEM.

To investigate the effects of NS and NP binding on viral replication, we first transfected HeLa cells with NSs and NS mutants, NSsW61Y plasmids, and then used a Western blot to examine changes in the NP protein level after infection with SFTSV at a multiplicity of infection (MOI) of 0.5 for 24 h. The result showed that endogenous NP protein accumulated in NS protein-overexpressing cells compared to NSsW61Y-overexpressing cells (Figure 1A,B). In our previous report, we confirmed the interaction between NP and NSs and determined that the 61st position of NS is important for the binding of these proteins. Furthermore, viral replication levels were affected by the NS mutants, which was verified with quantitative real-time polymerase chain reaction (qRT-PCR) and the 50% fluorescent antibody infectious dose (FAID_50_) [16]. It has been reported that NSs and NP are involved in autophagosome formation and replication [12,17,18]. Therefore, we further explored whether these viral proteins alter LC3 and p62 expression. Western blot analysis showed that overexpression of each viral protein after infection significantly decreased the protein levels of endogenous LC3I, LC3II, and p62 but not the level of NP (Figure 1C,D). It was also evident that the effect of the viral protein was much more profound on LC3II expression levels than those of LC3I. These results indicated that the wild-type NSs may be important in autophagosome formation.

### 2.2. Autophagosome Marker Levels Are Downregulated by NSsW61Y Expression

**Figure 2:**
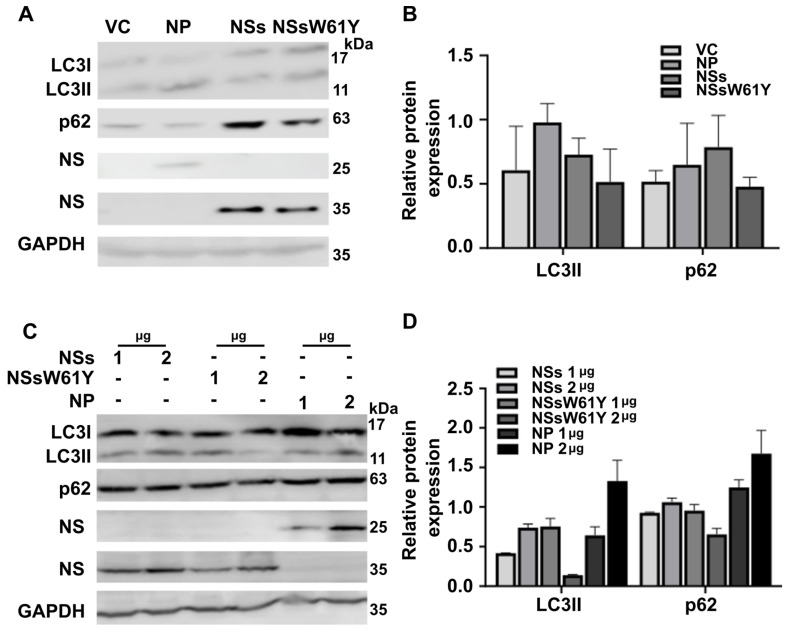
Dose-dependent downregulation of autophagosome marker levels by NSW61Y expression. (**A**) HeLa cells were overexpressed by transfection with each recombinant plasmid, and the levels of autophagosome-related genes were analyzed by Western blot assay. (**C**) Plasmids containing each gene were transfected into HeLa cells in a dose-dependent manner for 24 h. After cell lysis, the target protein expression was examined by Western blot. (**B**,**D**) Using Image J software, the quantitative expression levels of P62 and LC3II were normalized by GAPDH. The mean band density ±SEM is utilized for displaying results from independent investigations.

To investigate the effect of each viral protein, we exogenously overexpressed each recombinant plasmid in HeLa cells and confirmed the expression difference of the representative markers p62 and LC3 in the autophagosome (Figure 2A,B). Observations were conducted with and without SFTSV infection. After overexpressing each protein in a dose-dependent manner in HeLa cells, the levels of LC3 and p62 expression were measured by Western blot. The results showed that LC3II and p62 expression gradually decreased with the increase in NS mutant expression compared to the expression of NP and NSs (Figure 2C,D) in the presence of a viral infection. These findings revealed that the decrease in autophagosome-related factors observed during viral infection is the same as that of the viral protein overexpression. Thus, NS mutants that did not bind to NP inhibited autophagosome formation and consequently suppressed replication.

Comparing LC3I (17 kDa) and LC3II (11 kDa) expression, more profound suppression was observed in LC3II, the lipidated form of LC3I that was ready to integrate into the phagosome membrane.

### 2.3. Effect of NSsW61Y on Autophagy Related to the Lysosome-Dependent Degradation Pathway

**Figure 3:**
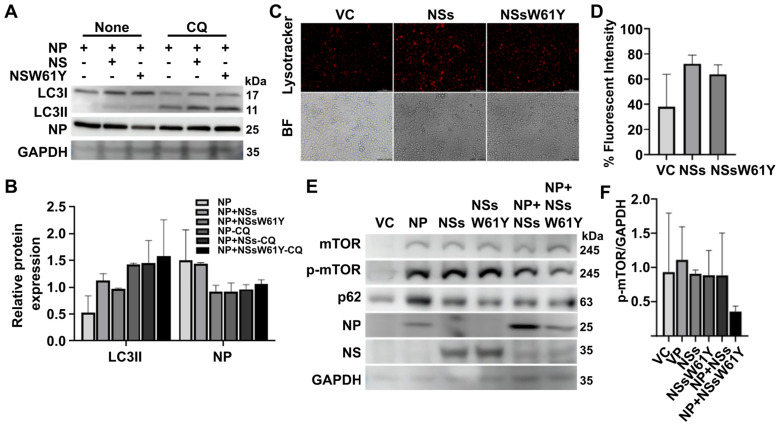
Effect of NSW61Y on autophagy-related to the lysosome-dependent degradation pathway. (**A**,**B**) Transfected HeLa cells were infected with SFTSV at an MOI of 0.5 for 24 h. Following infection, cells were treated with CQ at 100μM for 6 h, and cell lysates were examined by Western blot and analyzed the expressed protein levels of LC3II/GAPDH and P62/GAPDH. (**C**,**D**) Cells transfected for 24 h were incubated with LysoTracker deep red for 1 h before being processed for fluorescence microscopy. Quantitative analysis of fluorescence levels are illustrated and the independent experiments are presented as a mean fluorescence intensity ± SEM. (**E**,**F**) HeLa cells were co-transfected with recombinant plasmids of NP, NSs, NSsW61Y, NP-NSs, and NP-NSsW61Y, respectively. Cell lysates were evaluated through a Western blot. Representative quantitation of p-mTOR level was normalized by GAPDH and data from independent experiments are presented as a mean band intensity ±SEM.

We compared the expression of elements associated with autophagosome formation by chloroquine (CQ), an inhibitor involved in autophagosome and lysosome fusion, with the impact of viral proteins on the decomposition process through lysosome binding following autophagosome formation [19,20]. In addition, NSs and NSsW61Y were co-transfected with NP in SFTSV-infected cells and treated with CQ for 6 h to confirm the difference in binding with NP. Treatment with CQ significantly increased the accumulation of LC3-II and NP in NSsW61Y-transfected cells with NP. In contrast, untreated cells tended to decrease both factors (Figure 3A,B). LysoTracker was used to measure the efficiency of autophagosome/lysosome fusion in living cells as well as to detect variations in vesicle pH because it is a fluorescent dye that preferentially accumulates in acidic intracellular organelles [19,21,22] Previous research examined the possibility of the partial colocalization of autophagosomes and lysosomes tagged with LysoTracker Red [12]. In HeLa cells transfected with NSs and NSsW61Y, red fluorescence was stronger in NS-overexpressing cells than in NSsW61Y-expressing cells compared to the control group (Figure 3C,D).

### 2.4. The Relationship between SFTSV NSsW61Y and mTOR-Dependent Autophagy

Under normal conditions, mTOR acts as a negative regulator of autophagy through phosphorylation of downstream effector proteins [23,24,25]. However, when mTOR is inactivated, phosphorylation of downstream factors is inhibited, leading to induction of autophagy. Thus, mTOR-dependent signaling is crucial in regulating autophagy. Previous studies revealed that SFTSV infection decreased the phosphorylation of mTOR and the downstream effector unc-51-like kinase 1 (ULK1) [17,18]. Therefore, we also examined the changes in regulatory factors according to the presence or absence of viral proteins and their binding. The effect of NSW61Y-inhibited NP binding on autophagy formation was examined via the mTOR pathway, a classical regulatory pathway. Transfection and Western blot assay showed that SFTSV NSsW61Y expression indeed reduced conversion of mTOR to phosphorylated mTOR compared with the control, showing that NSsW61Y has a potential autophagy regulatory function via the mTOR axis (Figure 3E,F).

## 3. Discussion

Since the first case reported in China in 2009, SFTSV has been a significant public health concern on a global scale. Given that there is no cure for the infection in a clinical context, many studies have focused on understanding the infection mechanisms as the first step in determining an effective therapeutic approach. For decades, autophagy has been known for its role in antigen processing and presentation to the host immune system, outlining a well-conserved mechanism for preventing bacterial and viral diseases by mounting innate and adaptive immune responses [15,26]. These miniscule, intracellular double-membrane structures can transport viral nucleic acids and viral antigens and eliminate viruses by a process known as xenophagy [27]. In contrast, some viruses have developed to modulate autophagy for their advantage during viral replication and dissemination. Virus species such as HCV prevent IFN-I production and promote autophagy via protein accumulation, aiding in avoidance of innate immune responses and proliferation [14,28]. Human herpes virus destroys autophagy machinery to prevent activation of autophagy-related upstream signaling cascades [29]. Studies have demonstrated that the EBV promotes the maturation and export of viral particles, exploits autophagic vesicles as assembly sites, and effectively escapes from the lysosomal degradation of viral components [30,31,32].

Recent evidence related to SFTSV infections has demonstrated that it is also involved in autophagosome modulation during viral replication, especially when employing NSs and NP. Sun et al. have observed an increase in autophagy in Vero cells during SFTSV infection and found that NSs colocalized with LC3B protein [33]. Yan et al. have reported that SFTSV NPs targeting BECN1 induced host cell autophagy, and that autophagic vesicles facilitated virus assembly and egress [17]. This complexity and the importance of studying the mechanism of viral infection have been demonstrated in the relationship between host autophagy and viral infection. Secretory autophagy is known to promote viral maturation, egress, and cell–cell spreading and is initiated by SFTSV infection. In addition, the SFTSV infection did not exhibit a cytopathic effect, similar to some other viruses such as the SARS coronavirus [34]. Instead of merely observing a static state, the study revealed a dynamic morphological transformation (Supplementary Figure S1) over two days, marked by the emergence of multinucleated cells and a sharp increase in the concentration of the virus by the third day after infection. This phenomenon suggests a potential link between secretory autophagy, facilitated by cell fusion, and the pathogenicity of SFTSV. Nevertheless, the precise mechanism by which SFTSV influences autophagy remains enigmatic and warrants deeper inquiry. Recognizing the pivotal roles of NSs and NP in SFTSV replication and potential modulation of the autophagy process, our investigation elucidated the interplay between these two proteins and their subsequent impact on the autophagy pathway.

In our previous investigation, we determined that the 61st amino acid, tryptophan, of NSs plays an important role in the binding of NSs and NP [16]. It was confirmed that viral replication was decreased when the binding of these two proteins was inhibited. Hence, in this study, we further analyzed the effect of tryptophan-to-tyrosine NS mutation on the role in autophagosome modulation. In the initial step, we observed lower levels of expression of the autophagosome-related proteins LC3 and p62 in response to the exogenous expression of wild-type NSs and mutant NSsW61Y in HeLa cells (Figure 1A). Western blot images suggested a more profound suppression of LC3I and II bands, indicating the potential for transcriptional-level regulation of these proteins by SFTSV virulence factors. Furthermore, NSsW61Y could implicate an LC3I lipidation event that occurred during the formation of the phagocytic membrane, owing to the more pronounced suppression of LC3II expression observed in the Western blotting results (Figure 1C). Expression inhibition of LC3 and p62 proteins by NSsW61Y mutation was evident in HeLa cells with and without SFTSV infection (Figure 2A,B). We observed a more pronounced dose-dependent suppression of these proteins during SFTSV infection (Figure 2C,D), indicating the significance of the NSs and NP interaction on autophagosome formation.

During phagosome formation, the LC3 protein is cleaved into two subunits, LC3I and LC3II. The LC3II then undergoes a lipidation process by conjugating with phosphatidylethanolamine (L3II) and is subsequently integrated into the phagosome membrane. To further investigate autophagosome modulation by NSs and NP, we investigated the fate of LC3 marker expression when autophagosome formation is disrupted by 100 mM CQ. Chemical compounds such as CQ, 3-methyladenine, bapilomycine, and rapamycin have been known to inhibit autophagy and have demonstrated clinical significance as treatment for a variety of cancers and rheumatoid arthritis. Suppression of autophagosome formation by 100 mM CQ results in a reduction in LC3I protein levels, but both LC3I and LC3II proteins are present under conditions involving the exogenous expression of NP, NP with NSs, and NP with NSsW61Y. In the absence of CQ, a clear reduction in LC3II levels and enhanced LC3I protein levels were evident. This might indicate that SFTSV NSs and NP can influence the LC3I lipidation process thus positively regulating phagocytic membrane formation. Furthermore, fluorescent tracking of lysosome activity with LysoTracker stain reveals a reduction in fluorescent signal intensity (Figure 3C,D) when HeLa cells are transfected with NSsW61Y. Higher levels of fluorescence in NS wild-type transfected cells indirectly suggest that the SFTSV NSs are promoting lysosome fusion as a means of viral egress during the late phase of autophagy.

It is also evident that NS and NP proteins play a role in autophagy-related signaling pathways via mechanistic target of rapamycin (mTOR) modulation. Activation of mTOR (more precisely mTORC1) suppresses catabolic processes such as autophagy. Hence, viruses such as SFTSV compose mechanisms to suppress mTOR function as a means to promote autophagy by dephosphorylating mTOR. Mechanistically, the type I PI3K–AKT–mTOR signaling pathway is reduced during stress conditions, while the type III PI3K-–Vps34–Beclin1 complex is activated. mTOR inhibition dephosphorylates and activates the ULK1–ATG13–FIP200–Atg101 complex, which triggers autophagy initiation. Next, p62 acts as an adaptor/receptor for selective autophagy and as a signaling hub for mTOR activation on lysosomes and the Keap1–Nrf2 pathway on autophagic cargos. Here, we found that NSsW61Y regulates mTOR de-phosphorylation (Figure 3E). Therefore, a thorough understanding of the relationship between autophagy and SFTSV is necessary for determining whether the interactions of mTOR with the binding of the SFTSV viral proteins might influence downstream signaling axes related to mTOR. Although further investigations are needed, these data suggest that NSs may be responsible for processes such as ubiquitination so that SFTSV can use them for egress.

The findings of the current study highlight the essential role of SFTSV NS and NP proteins during viral replication and dissemination by intricate modulation of autophagy. It affects multiple pathways, such as autophagosome membrane formation, lipidation of LC3 proteins, lysosome fusion, and its phosphatase effect on mTOR proteins. Undoubtedly, further investigations are essential to elucidate the complete picture of SFTSV activity in infected cells. The insight gained by the current study might offer a pathway that uses NSs and NP as potential targets for therapeutic interventions against the challenging disease SFTS in humans.

## 4. Materials and Methods

### 4.1. Cell Culture and virus Propagation

Human Embryonic Kidney 293T cells (HEK 293T, ATCC CRL3216, Manassas, VA, USA), HeLa cells (ATCC CCL-2), and African green monkey kidney cells (Vero E6 cells, ATCC CCL-81) were cultured in Dulbecco’s Modified Eagle’s Medium (Gibco, North Andover, MA, USA), supplemented with 10% fetal bovine serum (Gibco) and 1% antibiotic–antimycotic solution (Gibco), at 37 °C in a humidified atmosphere with 5% CO_2_. The SFTSV strain KADGH (NCCP43261) was obtained from the National Culture Collection for Pathogens (NCCP, Chungbuk, Korea), propagated in Vero E6 cells, and maintained at 37 °C in a 5% CO_2_ atmosphere. The cell culture supernatant was collected 72 h after infection and centrifuged at 1000× *g* for 10 min at 4 °C to remove cell debris. Virus titers were determined using the FAID_50_ assay [35] and stored at −80 °C until further use. All experiments involving SFTSV were conducted in compliance with biosafety level 3 laboratory protocols following the guidelines of the Jeonbuk National University Biosafety Committee (JBNU2019-01-001-001) at the Korea Zoonosis Research Institute (KOZRI, Iksan, Jeonbuk, Korea).

### 4.2. Antibodies and Reagents

Primary antibodies, including LC3 (Cat# 4108), p62 (Cat# 5114), mTOR (Cat# 2983), and phosphorylated-mTOR-Ser2448 (p-mTOR, Cat# 5536), were procured from Cell Signaling Technology (Beverly, MA, USA). GAPDH antibody (Cat# sc-47724) was obtained from Santa Cruz Biotechnology (Dallas, TX, USA). Rabbit antisera to SFTSV NP and NSs were prepared as previously described [36,37]. The secondary antibodies of HRP-conjugated goat anti-rabbit IgG (Cat# 4030-05) and goat anti-mouse IgG (Cat# 1030-05), which were used in the Western blotting analysis, were purchased from Southern Biotech (Birmingham, AL, USA). Amersham ECL detection reagent was utilized for membrane development and was acquired from Cytiva (Marlborough, MA, USA). CQ, which was used to treat cells for 6 h before harvest, was obtained from Sigma-Aldrich (Saint Louis, MO, USA).

### 4.3. Plasmids and Transient Transfection

Open reading frames encoding NP, NSs, and NSsW61Y were amplified by polymerase chain reaction from synthesized genes and subsequently cloned into the expression vector pcDNA3.1 [16]. For transient transfection experiments, Lipofectamine™ 3000 Transfection Reagent (Invitrogen, Thermo Fisher Scientific, Inc., Waltham, MA, USA) and polyethyleneimine reagent (PEI, Sigma-Aldrich) were utilized as described previously [38]. Briefly, DNA transfections were performed according to the manufacturer’s instructions for each reagent. Transfected cells were then incubated under appropriate conditions for the desired duration of the experiment. This method ensured efficient delivery of the plasmids encoding NP, NSs, and NSsW61Y into the target cells, allowing investigation of their roles in cellular processes and viral replication.

### 4.4. Western Blot Analysis

HeLa and 293T cells were seeded into 6-well plates at a density of 1 × 10^6^ cells/well and transfected with pcDNA3.1, pcDNA-NP, pcDNA-NSs, or pcDNA-NSsW61Y at 1 μg/μL concentration using Lipofectamine™ 3000 Transfection Reagent or PEI. The transfected cells, 24 h post-transfection, were infected with SFTSV at a MOI of 0.5 for 24 h. Subsequently, the cells were washed with tris-buffered saline and lysed using radioimmunoprecipitation (RIPA, Bioseasang, Seongnam, Korea) buffer containing a protease inhibitor cocktail (Thermo Fisher Scientific). Following centrifugation at 13,000 x *g* for 15 min at 4 °C, cell lysates were subjected on the 6%, 10%, 12%, and 15% sodium dodecyl sulfate–polyacrylamide gel electrophoresis (SDS-PAGE), respectively, and transferred to a polyvinylidene difluoride membrane (Millipore, Billerica, MA, USA). The membranes were blocked with 5% bovine serum albumin (BSA, Geneall, Seoul, Korea) for 1 h and then incubated overnight at 4 °C with primary antibodies diluted in 1% BSA buffer, including LC3 (1:1000), p62 (1:1000), mTOR (1:1000), phosphorylated-mTOR (1:1000), GAPDH (1:1000), and NP and NS hyper-immune sera (1:1000). After washing with tris-buffered saline with tween 20 (TBSt), the membranes were incubated with goat anti-rabbit HRP-IgG or goat anti-mouse HRP-IgG secondary antibodies at a dilution of 1:10,000 for 1 h at room temperature. Finally, the membranes were developed using Amersham ECL detection solutions (Cytiva) and visualized using an ImageQuant 800 imaging system (Cytiva). The protein band density was analyzed with Image J (version 1.53 k, US National Institutes of Health, Bethesda, MD, USA). Data from independent experiments are presented as a mean band intensity ± SEM. This method enabled the detection and quantification of target proteins and facilitated the analysis of their expression levels in response to SFTSV infection or protein transfection.

### 4.5. LysoTracker Deep Red Staining and Immunofluorescence Microscopy

LysoTracker Deep Red (Invitrogen) staining was performed according to the manufacturer’s protocol. HeLa cells were seeded in 12-well plates at a density of 1 × 10^5^ cells /well. Cells were co-transfected with the pcDNA-NP plasmid along with each NS and NSsW61Y expression plasmid using Lipofectamine™ 3000 Transfection Reagent. Following transfection, the medium was removed, and the cells were incubated with prewarmed serum-free medium containing 50 nM LysoTracker Deep Red dye for 1 h. Subsequently, the cells were examined using a Leica Fluorescence Microscope (Leica Biosystems, Wetzlar, Germany) to visualize lysosomal compartments labeled with the LysoTracker dye. This method allowed assessment of lysosomal morphology and distribution in response to transfection with SFTSV NP and NSs. The observed changes in lysosomal dynamics provided insights into the cellular processes influenced by SFTSV proteins and their potential roles in viral pathogenesis.

## Data Availability

Data are available from the authors upon reasonable request.

## Supplementary Materials

The following supporting information can be downloaded at https://www.mdpi.com/article/10.3390/ijms25126394/s1.
