# Supplementary material for: The Effect of Tryptophan-to-Tyrosine Mutation at Position 61 of the Nonstructural Protein of Severe Fever with Thrombocytopenia Syndrome Virus on Viral Replication through Autophagosome Modulation
Source: Int J Mol Sci. 2024 Jun 10;25(12):6394. doi: 10.3390/ijms25126394 (PMC11203599; doi:10.3390/ijms25126394)
Supplement: Supplementary file 1 [file ijms-25-06394-s001.zip › ijms-2960237-supplementary.pdf]

Supplementary Figure S1

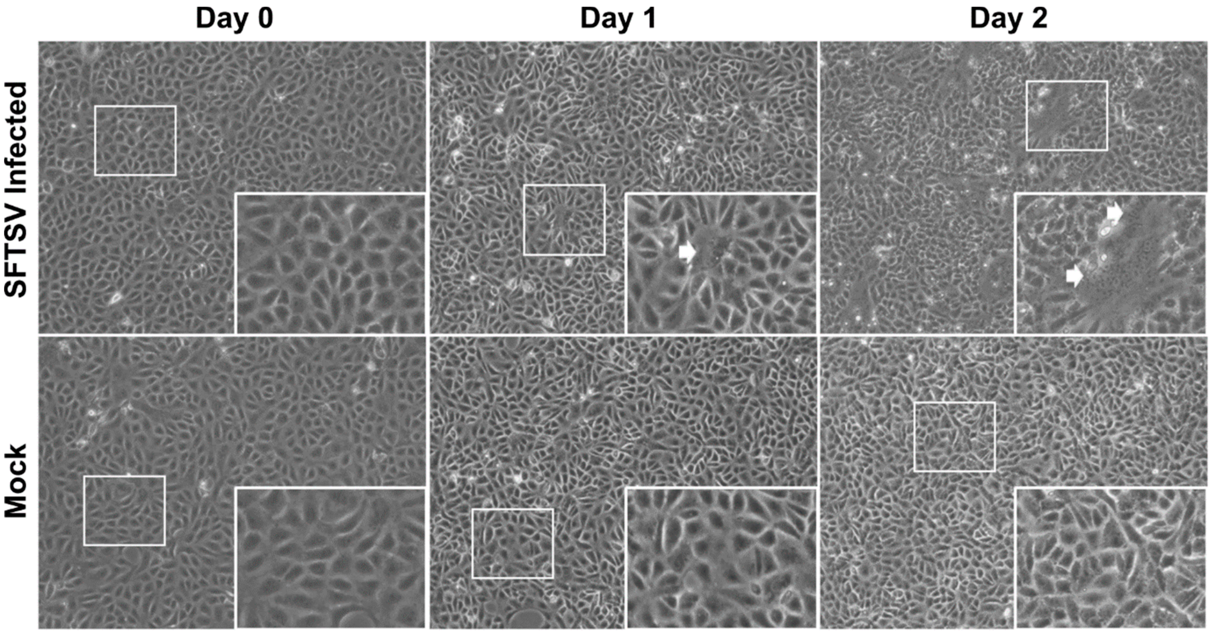

Supplementary Figure S1. Changes in Vero Cells after SFTSV Infection. Images depict alterations observed in Vero cells following SFTSV infection. Two days post-infection, cell fusion and syncytial development were observed. By day three post-infection, there was an increase in the frequency of multinucleated giant cells.
